# Principal Components and Hierarchical Cluster Analyses of Trace Metals and Total Hydrocarbons in Gills, Intestines and Muscles of *Clarias gariepinus* (Burchell, 1822)

**DOI:** 10.1038/s41598-020-62024-9

**Published:** 2020-03-20

**Authors:** Patrick Omoregie Isibor, Tunde O. Thaddeus Imoobe, Alex Ajeh Enuneku, Paul Akinniyi Akinduti, Gabriel Adewunmi Dedeke, Theophilus Aanuoluwa Adagunodo, Dorcas Yemisi Obafemi

**Affiliations:** 1 Department of Biological Science, Covenant University, P.M.B. 1023, Ota, Ogun State Nigeria; 2 Department of Animal and Environmental Biology, University of Benin, P.M.B. 1154, Benin City, Nigeria; 3 Department of Pure and Applied Zoology, Federal University of Agriculture, P.M.B. 2240, Abeokuta, Ogun State Nigeria; 4 Department of Physics, Covenant University, P.M.B. 1023, Ota, Ogun State Nigeria; 5 Department of Environmental Management and Toxicology, University of Benin, P.M.B. 1154, Benin City, Nigeria

**Keywords:** Animal physiology, Biomechanics, Environmental impact

## Abstract

The aim of the study was to comparatively analyze the interrelationships among iron (Fe), manganese (Mn), zinc (Zn), copper (Cu), lead (Pb), cadmium (Cd), chromium (Cr) and total hydrocarbons (THCs) in the gills, intestines and muscles of *Clarias gariepinus* collected from Osse River, Nigeria, between the periods of April, 2013 to September, 2014. The trace metals in the fish tissues were analyzed using Atomic Absorption Spectrophotometer (AAS, Philips model PU 9100), while total hydrocarbons were analyzed using High Performance Liquid Chromatograph (HPLC,Prominence Dual brand from HGE) equipped with a detector Shimadzu UV-Visible (UV-Vis Prominence SPD 20 A). The concentrations of trace metals and THCs in the tissues were subjected to principal component analysis (PCA), in conjunction with hierarchical cluster analysis (HCA), backed up by correlation analysis (CA). In the most prioritized component among the hierarchies of contaminants, characterized as principal component 1, results of communality extractions and rotated component matrices revealed the order of contaminants was Mn > Cu > Zn > Fe > Cr in the intestines, Cr > Cu > THCs > Mn > Fe in the muscle, while Pb > Cr > Fe > Mn was the order in the gills of the fish. Iron inhibited accumulation of the other trace metals in the gills, where its threshold of essentiality was maximal. Noteworthy is the fact that Mn and Cu were the most active components in the muscle and concurrently of excess concentrations in the tissue, which is the major edible part of fish, and constitutes its main body weight, hence holds its nutritional and economic values. High level of variability which occurred in the toxicant profile across the tissues of *C. gariepinus* is a function of uptake route, varied organ functions and specificity of tissue permeability of the compared organs. The study demonstrated variability in organ accumulation capacity and toxicant’s competitiveness irrespective of bioavailability. The study provides data useful for future ecotoxicological studies and safety of consumers of the fish.

## Introduction

Ecotoxicological studies aimed at evaluating the concentrations of toxicants in environmental matrices provide information such as the levels of toxicants in tested media, relative to set standards by regulatory agencies. Toxicokinetcis and toxicodynamics of xenobiotics such as trace metals and hydrocarbons in aquatic fauna are influenced by numerous intrinsic and extrinsic factors^1–3^. Furthermore, toxicants may exhibit synergistic, antagonistic or supra-addictive interactions. Hence a seemingly low concentration of a given toxicant may elicit unexpectedly high toxicity effect in the presence of another toxicant. The exposed biota may also present prioritized absorption routine to the bioavailable toxicants^1,4–6^. The net effects of the factors presented by the contaminants and the biota may favour the absorption of non-essential metals and on the other hand impair absorption of essential metals in dietary constituents^7^. Enhancement of non-essential metal uptake may elicit toxicity effects while impairment of essential metal may simultaneously result in malnutrition. Studies have shown that chemically similar metals may share a pathway for absorption, thus resulting in competition for absorption by cells of mucosa^7–9^. Essential elements are required in diet for optimal growth, functioning and sustenance of the internal environment^10^.

Iron deficiency may lead to anemia; the most common nutritional deficiency in the world^11^. Anemia is a condition of low hemoglobin; a protein responsible for oxygen transportation. Excess iron in human may result in symptoms such as profuse vomiting, diarrhea, abdominal pains, and dehydrations. In the fish, excess iron may elicit histological alterations, characterized by gill tissue necrosis, impairment of the excretory function of the gills and elevated activity of transaminases in the blood plasma. Studies have shown that excess iron in fish may also result in dystrophic alterations and injury in the parenchymatous tissues such as the kidney, liver, and spleen^12^. Fe^2+^ may bind to the alkaline gill surface and oxidize to insoluble Fe^3+^. The insoluble iron could cover the gill fringes, thereby inducing epithelial damage, thus disrupting respiration^13–15^. Iron bacteria thrive in water bodies with high Fe content, particularly in low pH and temperature. The iron bacteria (*Acidithiobacillus sp*. and *Leptospirillum sp*.) derive energy and proliferate by oxidizing dissolved ferrous iron, or the less available manganese^13^. The by-product of the reaction is insoluble ferric oxide or manganese dioxide. The bacteria create colonies on gill surfaces thereby inflicting histological damage on the gill architecture, hence compromising the structural and functional integrity of the organ.

Zinc deficiency is associated with poor growth, hampered replacement of worn-out tissues, and impaired immune response^16,17^. Ecological Zn concentration may rise above permissible levels through anthropogenic activities such as mining, coal and waste combustion and steel processing. Overdose of Zn may cause respiratory disorders, arteriosclerosis, disrupted protein metabolism^16^. McRae *et al*.^18^ exposed galaxiid fish, *Galaxias maculatus* to environmentally-relevant concentrations of Zn (8, 270 and 1000 μg/L) over 96 h to determine the impact on ionoregulation, respiration, oxidative stress and bioaccumulation of Zn. They observed increase in catalase activity and lipid peroxidation at 1000 μg/L. They also observed inhibition of calcium influx, but promotion of sodium influx, at 1000 μg/L. The sub-lethal changes induced by Zn exposure in the fish appeared to be conserved relative to other better-studied species^4,8,9,18,19^.

Approximately 91% of environmental manganese is released unto soil from land disposal of manganese-containing wastes. Manganese in water settles onto suspended sediment particulates. Anaerobic groundwater often contains elevated levels of dissolved manganese^17^. The divalent form of manganese: Mn^2+^ predominates in most water at pH 4–7. More highly oxidized form of manganese may occur at higher pH values or result from microbial oxidation^20^. Manganese can accumulate in lower organisms, such as phytoplankton, algae, mollusks and fish, but does not readily accumulate in higher organisms. In nature, biomagnification of manganese in food chains is often at low rates. However, extreme cases of exposure to manganese may result in neurological disorders, brain damage, reproductive, developmental toxicity^21^, lung, liver and vascular tissue injuries^22^. Also plants are affected by highly toxic concentrations of manganese that can cause swelling of plant cell walls, withering and brown spots on leafs.

Copper deficiency is a state of insufficient serum copper level, which results in hematological^23^ and neurodegenerative syndromes such as myelopathy, and optic neuropathy^24,25^. Conversely, when in excess, copper is one of the most toxic metals to aquatic organisms and ecosystems^21^. Copper is moderately soluble in water and binds easily to sediments and organic matter^26–28^. Hence, it is readily bioconcentrated in pelagic and benthopelagic fishes of watersheds polluted with copper-associated contaminants^29^. Exposure to high copper concentrations disrupts fish’s sense of smell, thereby affecting their feeding, reproduction and escape from predators. Sandahl *et al*.^30^ pointed out that copper may interfere with olfactory functions of salmons and therefore disrupt olfactory-mediated behaviours, such as homing, which is important for the survival and migration of the fish. Therefore dwindling population of some fish species may be partly linked to copper pollution of the water bodies.

In aquatic ecosystems the susceptibility to cadmium may varies greatly among aquatic organisms. Salt-water organisms are known to be more resistant to cadmium poisoning than freshwater organisms^31^. Cadmium accumulates in the kidney, liver, and gills of freshwater fish^32,33^. Cadmium accumulation in these organs is mediated by binding molecules called metallothioneins^4,33^. Dallinger *et al*.^33^ posited that alkalinity plays a role in where cadmium and other metals accumulate and to what extent. A study demonstrated that the liver metallothionein was dominated by copper and zinc in spite of high cadmium levels in the kidney^33^. As a result, in aquatic habitats that have low alkalinity, fish may uptake environmental cadmium at marked levels. Levit^4^ submitted that in the gills, cadmium burdens can result from both dietary and waterborne cadmium exposure^32^. Hence, gill burdens are not diagnostic of the exposure route^32,34^.

Fuel combustion, industrial processes and solid waste combustion are anthropogenic sources of Pb in the environment. It can end up in water and soils through corrosion of leaded pipelines in a water transporting system and through corrosion of leaded paints. The environment receives substantial amount of Pb from the use of Pb containing additives used for gasoline treatment. Martinez *et al*.^35^ *Prochilobus lineatus* acutely exposed to 24 mg/L of Pb for 96 h elicited histological gill lesions, temporary disruption of sodium regulation, reduced protein, and lipids, a dose-dependent oxidative stress and histopathological lesions such as epithelial lifting, hyperplasia and lamellar aneurism. They attributed the changes observed to defense responses of the fish which is based on increase of the distance between the pollutants in the ambient water and the fish plasma^35^.

Anthropogenic sources of chromium include mining, improper waste disposal, and fuel combustion^36^. Several studies have shown evidences of chromium toxicity in various fish species^11,23,32,37,38^. Reduced blood clotting^32^, decrease in white blood cells, red blood cells counts and hemoglobin concentration was observed in *Tilapia sparrmanii* exposed to chromium concentration of 0.0098 mg/L at pH 7.4–9.0^11,37^. Khangarot *et al*.^23^ detected increases in spleen to body ratio, hemoglobin, white blood cells, packed cell volume (PCV), splenocytes, and antibody production, hence susceptibility to bacterial infection, when *Saccobranchus fossilis* were exposed to 0.1–3.2 mg/L chromium. *Periophthalmus dipes* exposed to chromium 5–15 mg/L exhibited decrease in ion-dependent ATPase activity^29^. Vutukuri^38^ demonstrated decrease in glycogen content, total lipid content, and protein contents of muscle, gill and liver of *Labeo rohita* at 39.4 mg Cr L- (96 h-LC50). *Carassius auratus* exhibited decrease in cell viability and increase in reactive oxygen species when exposed to 250 µM Cr^39^. While *Colisa fasciatus* showed decrease in liver glycogen content^37^.

Studies have shown that high concentrations of Fe in solution can negatively affect Zn absorption^11^. Manganese-induced alterations in metal absorption and competition for metal transporters and regulatory proteins were also reported by Foster^7^. An inverse relationship between hepatic manganese and iron concentrations has been previously observed^6,19,28^. This is due to the fact that manganese and iron transports are regulated by transferrin receptor and divalent metal transporter.

Fatty fish, such as *C. gariepinus* are capable of accumulating hydrocarbons in their tissues to concentrations much higher than in the water and sediment of their habitat^40–43^. This owes to high biomagnification capacity of the fish, owing to its high lipid content. The lipophilic nature of hydrocarbons also plays a role in its accumulation in fish^43–46^. Furthermore, hydrocarbons readily adsorb on particulates which aid their precipitation to the bottom sediment, thus further exposing demersal fish like catfish and other bottom dwellers to the toxicants^46–48^. For example, 14–315 ng/g polycyclic aromatic hydrocarbons (PAHs) was detected in demersal fish^41–44^, 54–2803 ng/g was detected in mollusk^22^, 52–1600 ng/g in crustacean^49^, and 284–4665 ng/g in algae^48^. All these levels are of ecotoxicological concern. Law and Klungsoyr^22,43^ earlier posited that hydrocarbons are notable for high affinity for sediments and lipid tissues of fish, hence may pose carcinogenic risk to humans who consume such fish.

Studies of interactions among toxicants have shown that mere comparison of levels of toxicants with regulatory standards may not provide satisfactory backdrop for safe decisions. Hence, such generalized approaches used in toxicological studies are often concluded with recommendations for further studies to ascertain the actual constituents in environmental media which may be responsible for the contamination^46,47^.

Principal component analysis (PCA) could be employed as a statistical tool to analyze the interactions among a set of toxicants, in order to assign case-specific grades to the toxicants according to their toxicity potentials. Hierarchical cluster analysis (HCA) may further depict the correlation in the interaction of the toxicants and their interrelationships within a given environmental medium. Previously, PCA and HCA have been widely applied in evaluation and classification of toxins^15,50–54^.

*Clarias gariepinus* was chosen as the candidate for investigation in this study due to its ecological abundance and economic significance to the populace. Moreover, the fish species has potential for toxicant accumulation due to its pelagic feeding habit and high adipose content which readily biaccumulates lipophilic contaminants^21^. The study was aimed at comparatively analyzing the interrelationships and priorities in the absorption of trace metals (Fe, Mn, Zn, Cu, Pb, Cd and Cr) and total hydrocarbons (THCs) in the gills, intestines and muscles of *Clarias gariepinus* using multivariate analyses. Multivariate analyses such as PCA and HCA may provide better understanding on the relative activities of the toxicants, beyond the bioaccumulation factors. These findings may add knowledge to the toxicokinetics and toxicodynamics of these toxicants.

## Materials and Methods

### Description of study area

**Figure 1:**
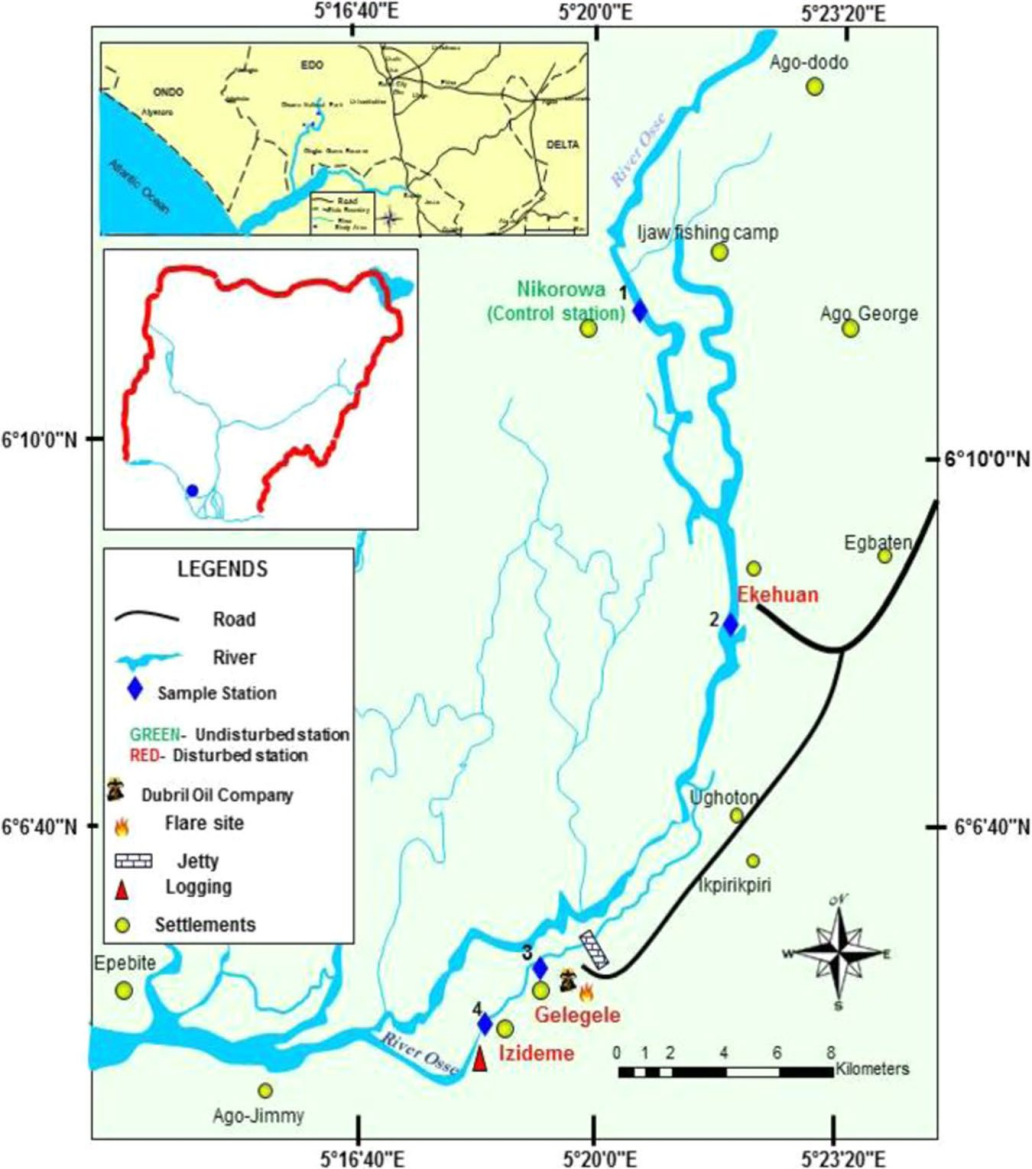
Map of the study area showing sampling stations. Map designed using QGIS software version 3.10.1 ‘A Coruña’ (QGIS Development Team^1^) URL: https://qgis.org/en/site/forusers/download.html#.

The study area is a stretch of Osse River (06° 13.432′N, 05° 20.826′E), between Benin River and Ughoton stream which transverses from Nikorowa, through Ekehuan and Gelegele to Iziedema communities. It is located in Osse North-East local Government Area of Edo State; within the tropical rainforest belt of Southern Nigeria (Fig. 1). It is an oligotrophic^55^ freshwater river with a thick tropical vegetation cover along its bank. The river is the major source of domestic water for the inhabitants of Gelegele and other communities in the catchment area. The vegetation of the study area comprises mainly of thick trunk trees amidst shrubs and grasses. Most of the plants which dominate the surrounding environment of the study area include; varieties of bamboo trees (*Bambusa sp*.), water hyacinth (*Eichhornia crassipes*) and palm trees (*Elaeis guineensis*).

An oil company named Dubri Oil Company Limited (DOCL) has a flow station close to the Gelegele section of the river; where it has produced about 7.778 million barrels of light crude oil (API 45–50) since its inception in August, 1987^47^. Logging activities were predominant in the forest within the catchment area. The logs were further sliced into marketable sizes at a saw mill at the bank of the river. Such logging activities may be a source of particulates, which may serve as adsorptive substrates to toxicants, thus aggravating fish exposure as they clog up the gills.

### Sample collection

Surface water samples were collected at the four (4) locations of distinct anthropogenic activities at the river. The water samples were collected in clean sampling bottles and transported immediately to the laboratory for analysis of the concentrations of the trace metals and THCs. The results were documented on monthly basis. A total of 72 samples of *Clarias gariepinus* representatives were collected at the four locations along the river course; making a total of 288 fish samples (118.3–218.5 g). The fish were collected by informed artisanal fishermen with the aid of fishing nets, baskets and hooks. They were preserved in a chest chamber at −4 °C, and taken to the laboratory of Animal and Environmental Biology, University of Benin, Nigeria for proper identification using manuals such as Idodo-Umeh^56^ and Olaosebikan and Raji^57^. These procedures spanned a period of April, 2013 to September, 2014.

#### Dissection of fish and excision of organ

The gills, intestines, and muscles of the fish were chosen on the basis of the significant roles the organs play in the animal. The gills were chosen for investigation being the main organ which actively interacts with toxicants in the aqueous phase. It also mediates ion regulation, by the help of the chloride cells. Furthermore, most restaurants do not discard the organ before processing; hence the toxicants accumulated in the gills might pollute the entire food. The intestines are highly adapted for maximal absorption of ingested nutrients alongside toxicants, owing to its large surface area offered by the numerous villi. The muscles of fish constitute its major nutritional and economic values^58^, hence needs to be screened for fitness of consumption.

To excise the organs, the fish was pinned onto a clean dissecting board with underside of the fish facing up. The fish was then dissected ventrally in a triangular pattern. A cross sectional incision traversed between the left and right pectoral fins and tapered at the anus. The intestine was located adjacent to the gonads; identified as the coiled organ which extends from the stomach to the anus. Afterwards, the operculum was removed to expose the gills which were collected in an appropriately labeled sampling bottle.

For convenience, the muscle tissue was obtained from a muscular part of the fish; between the pectoral fin and the dorsal fin. The epithelial layers of the fish were examined using a binocular dissecting microscope (American Optical Corporation, Model 570) and the layers were removed using a sterile surgical blade. The epidermis layer and the basal membrane beneath were carefully peeled off to avoid mechanical damage to the subsequent layers. Afterwards, the dermis was identified by 2 layers which include the stratum laxum and stratum compactum which were carefully removed to expose the subcutis. The subcutis was carefully scrapped off to expose the muscles. The muscles was then excised and kept in a properly labeled sampling bottle for tissue analysis.

#### Analysis of trace metals

##### Trace metals in fish tissues

The gills, intestines and muscles of fish were eviscerated using sterile blades. Wet weight (10 g) of each tissue was extracted using 20 mL of HNO_3_: HClO_4_ (5:1) at 105 °C for about 24 h^59^. The extract was made up to 25 mL with HNO_3_ (70%) and diluted with deionized water. Atomic Absorption Spectrophotometer (AAS, Philips model PU 9100) was used in reading trace metals concentration in mg/kg; wet weights. The meter was calibrated for each metal by dissolving 1 g analar grade metal salt in 1 L of distilled water according to the guidelines of Aderinola *et al*.^26^.

##### Trace metals in water samples

Ten (10) mL of water sample was put in a beaker and 2 mL nitric acid was added. The content of the beaker was then heated to evaporation and allowed to cool afterwards. The mixture was transferred into volumetric flask. It was then allowed to stand for 24 h, after when it was centrifuged until clear. The sample was screened for suspended solids which were filtered prior to analysis. The trace metals in the mixture were then read using an AAS at 250–350 Volts using the ABS knob.

#### Analysis of THCs

##### THCs in fish tissues

Extraction and analysis of THCs in fish tissues were performed according to International Standard Organization (2004). A sample of 2.5 g (wt weight) of fish intestine or reference sample was introduced into a centrifuge tube, we then added 10 mL of acetonitrile/acetone (V/V, 60/40). Whole tissue was homogenized by vortexing 30 sec and 5 min in ultrasonic bath before being centrifuged for 5 min at 4000 rpm. We removed the supernatant and transferred it into a conical tube and the solvent was heated at 35 °C. The extraction was repeated twice with 10 mL of acetonitrile/acetone. The extract was then purified on the cartridges of bonded phase C18 (Waters Sep Pack). Purification was done by adding 2 mL of acetonitrile/acetone are introduced into a conical tube containing the sample. The mixture was vortexed for 15 sec and centrifuged for 30 sec. The upper phase was transferred into a tube and the operation was repeated twice. The supernatants were transferred onto a C18 cartridge previously conditioned with 12 mL of methanol and 12 mL of acetonitrile. The elution was performed with 5 mL of acetonitrile/acetone at atmospheric pressure. The eluent was thereafter concentrated to 50 mg using a rotary evaporator at 35 °C. The purified extract was recovered in 1 mL of hexane. The tube was crimped and stored at −18 °C before analysis.

PAHs were determined using a High Performance Liquid Chromatograph (HPLC) (Prominence Dual brand from HGE) equipped with a detector Shimadzu UV-Visible (UV-Vis Prominence SPD 20 A), a pump (LC 20 AD Prominence Chromatograph), an automatic injector (Autosampler Prominence SIL 20AC) and a column (Column Prominence Owen CTO 20A) type Prevail C18 (15 × 4.6 mm × 5 microns). The cartridge temperature was 40 °C, flow rate was1mL/minute and at wavelength of 254 nm^41^.

##### THCs in water samples

Analysis of THCs in the water samples was carried out by extracting 1 L of water sample using an AccuBond ODS C18 SPE cartridge (Agilent p/n 188–1356). The cartridge (0.5 g) was conditioned by sequentially rinsing it with four 10-mL aliquots of dichloromethane (DCM), methanol, and two 10-mL aliquots of water (HPLC grade). Afterwards, 1-L of collected water sample was passed through the SPE cartridge at a flow rate of 2.5 mL/min using an automated solid phase extractor. The cartridge was then rinsed thoroughly with 10 mL of HPLC water and then dried with nitrogen for about 10 min. THCs was eluted from the cartridge with 5-mL DCM twice and then added together. The elute was then evaporated with a stream of nitrogen to a volume of 1 mL, and 3.0 mL of acetonitrile was added, which was then concentrated to a final volume of 1.0 mL (USEPA, 1990). THCs concentration was determined using a High Performance Liquid Chromatograph (HPLC).

#### Quality control and quality assurance measures

Before use, the dissecting instruments and sampling containers were pre-cleaned using 80% ethanol and sterilized 121 °C for 15 min, using a pressure steam sterilizer (Model: SM280E) by Surgifriend Medicals, England. One surgical blade was used per tissue sample, after when it was discarded safely. To avoid hand contamination of the samples, sterile laboratory gloves and nose masks were used throughout the experimental session. All readings were taken in triplicates to minimize error.

##### Validation of trace metals

Precision and accuracy of the AAS and HPLC methods were validated by repeating each procedure 3 times. Certified reference materials (CRM) by Federal Environmental Protection Agency^60^ were employed as a guide. The recovery rates ranged from 93–97%, relative standard deviation (SD) was <6% (determined by Microsoft Excel, 2010), indicating a high data integrity. The reference solutions used to obtain the calibration curves were prepared from a stock solution containing 1000 mg L–1 of each trace metal analyzed. The blanks and reference solutions were also analyzed using the same method that was applied to the samples. The concentrations were expressed in mg/kg.

The limits of detection (LOD) and the limits of quantification (LOQ) were calculated based on the standard deviation of 20 readings obtained for the analytical blanks and the slopes of the analytical curves (LOD = 3σ/slope and LOQ = 10σ/slope). The values (mg/kg) were 0.05–0.07 (Fe), 0.06–0.08 (Mn), 0.02–0.04 (Zn), 0.06–0.12 (Cu), 0.07–0.123 (Pb), 0.06–0.121 (Cd), and 0.043–0.127 (Cr).

##### Validation of THCs

The quantification of THCs was validated using reference guidelines of Ake *et al*.^41^ and readings of the HPLC were verified by the certificate (No. TPH-R3-SET, by AccuStandard). To ensure accuracy of equipment we conducted a study of the linearity of the calibration range. The LODs and LOQs were determined and further calculated the coefficient of variation for the tests of repeatability and reproducibility. The calculation of the percentage recovery for testing accuracy was also conducted. The linearity was tested between 0 and 10 μg/kg. The LODs and LOQs were calculated from the blank and analyzed by the HPLC. Limit of detection was calculated with 3 separate trials thus;

$${\rm{LD}}={\rm{mb}}+3\,{\rm{SD}}$$

While limit of quantification was calculated with 10 separate trials thus;

$${\rm{LQ}}={\rm{mb}}+10\,{\rm{SD}}$$

where mb = average concentration with the blank and SD = standard deviation of blank values.

#### Compliance statement

Fish used for the experiment were purchased fresh but lifeless from fishermen who provided the information on standard fishing techniques and handling procedures adopted. Effective collaboration with the fishermen was aimed at ascertaining quality control in the study. All experimental procedures were performed in accordance with standard scientific guidelines and regulations. Methods were carried out in accordance with relevant guidelines and regulations.

### Statistical analysis

The concentrations of the trace metals and THCs in the tissues of 288 fish samples collected over the period of 18 months were reported as mean values and standard deviation (SD), which were subjected to analysis of variance (ANOVA) to test for the significant differences. They were further subjected to Duncan Multiple Range (DMR) test at 95% confidence interval and probability level of 0.05 using SPSS (version 20). The Kaiser-Meyer-Olkin (KMO) analysis was conducted on the concentrations of toxicants in the tissues to assess the sampling adequacy and suitability for use in the study. The KMO output for the gills at 0.6 was declared as significant variance in data, attributable to the varied accumulation tendencies of the tissues and competition for sites among the toxicants. Furthermore, the sphericity of the data was analyzed using the Bartlett’s test which gave a *p-value* of 0.032 (significant), indicating that a factor/factors may be responsible for the significant variability detected in the data analyzed.

Although power calculation was not conducted for data in this study, the sample size being greater than 100 however guarantees reliable statistical power (Aschard *et al*.^61^, Jolliffe^23^). The robustness of the sample size most likely negates the inadequacies associated with natural factors^62^.

**Figure 2:**
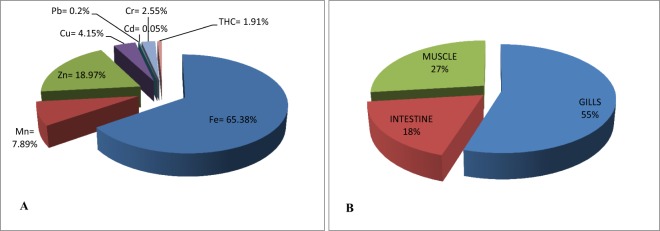
(**A**) Percentage composition of trace metals and total hydrocarbons in *C. gariepinus*. (**B**) Percentage distribution of trace metals and total hydrocarbons in tissues of *C. gariepinus*.

**Figure 3:**
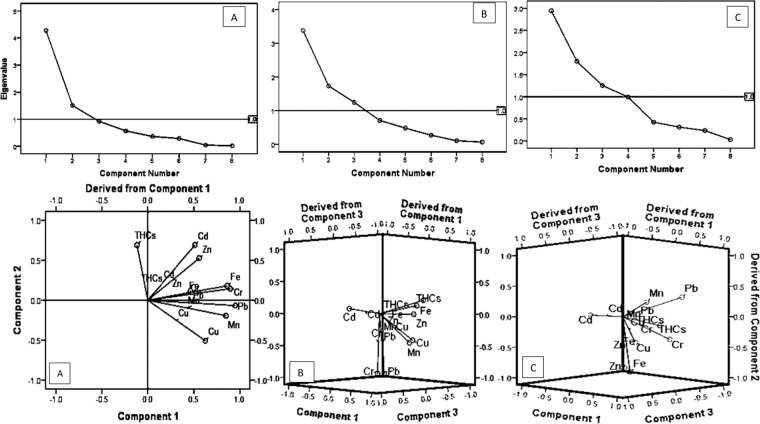
Scree plots showing principal components and benchmark Eigenvalues (I). Components plots in rotated space (II). Keys: Gills (**A**), Intestines (**B**), and Muscles (**C**) of *C. gariepinus*.

Principal component analyses were separately conducted for toxicant concentrations in the different tissues using SPSS (version 20) and verified by Microsoft Excel, 2010 (XLSTAT statistical software). Eigenvalues were obtained for the 288 readings of trace metals and THCs concentrations in the gills, intestines, and muscles of the fish samples through communality extractions. Eigenvalue 1 was adopted as the significant benchmark for the components. Components 1, 2, and 3 were above 1 in the cases of the intestines and muscles (see Fig. 2I,B,C respectively), hence were extracted for further analysis. Components 1 and 2 were extracted for the gills as they were above 1 as indicated by the horizontal line (see Fig. 3I,A). Afterwards, direct oblimin was used in analyzing the significant components obtained from the PCA, following oblique output from KMO and Bartlett’s analyses. The components were represented by a coordinate axis which was projected onto two (for 2 components as in the case of the gills) and three (for 3 components as in the cases of the intestines and muscles) dimensional graphs for ease of spatial visualization of the statistical distribution of the data. Exclusion of values was based on the position on the list and the coefficient display format was sorted by size.

For clarification of actual value, PCA was complemented with hierarchical cluster analysis (HCA) which was determined using average linkage hierarchical clustering to evaluate inter-relatedness of toxicant concentrations in the tissues and correlation analysis was employed to further ascertain the interrelationships among the toxicants analyzed.

### Ethical approval

Fish used for this study were fresh but lifeless, however all procedures used conform to standard scientific research guidelines. The source and pre-purchase handling of the specimens were thoroughly investigated for quality assurance purpose.

## Results

As presented in Table 1, concentrations of iron and total hydrocarbons in the gills of C. gariepinus exceeded the set standard limit of Federal Environmental Protection Agency (FEPA)^60^. They were also significantly higher than the concentrations in the intestines and muscles (*p* ≤ *0.05*). The concentration of Zn in the intestines exceeded the standard limit of FEPA and was significantly higher than the concentrations in the gills and muscles (*p* ≤ *0.05*). The concentrations of Mn and Cu in the muscle exceeded the regulatory limit and were significantly higher than the concentrations in the gills and intestines at *p* ≤ *0.05*. In the water, only the concentration of manganese was above the established limit. The concentrations of every other toxicant in the tested media were below the FEPA limits^60^.

**Table 1:** Concentrations (mg/kg wt w) of trace metals and THCs in gills, intestines, and muscles of *C. gariepinus* (Mean ± S.D).

Toxicant	Water	FEPA^60^ (Water limit)	Gills	Intestines	Muscles	FEPA^60^ (Tissue limit)	Gill BCF	Intestine BCF	Muscle BCF
Fe	1.25 ± 0.55	20	**181.8** ± **42.3**^**A**^	26.1 ± 7.8^B^	32.8 ± 18.7^B^	100	**145.44**	**20.88**	**26.24**
Mn	**0.62** ± **0.59**	0.5	0.41 ± 5.1^B^	0.5 ± 2.7^B^	**6.5** ± **2.5**^**A**^	1	0.66	0.81	**10.48**
Zn	0.66 ± 0.39	1	27.8 ± 6.8^B^	**88.9** ± **7.5**^**A**^	38.1 ± 5.5^B^	75	**42.12**	**134.7**	**57.73**
Cu	0.34 ± 0.48	<1	0.2 ± 2.6^C^	0.5 ± 1.4^B^	**4.6** ± **1.2**^**A**^	3	0.59	**1.47**	**13.52**
Pb	0.44 ± 0.45	<1	1.1 ± 0.2^A^	0.2 ± 0.03^B^	0.1 ± 0.03^B^	2	**2.5**	0.45	0.23
Cd	0.18 ± 0.18	<1	0.1 ± 0.02	0.1 ± 0.02	0.2 ± 0.09	2	0.56	0.56	**1.11**
Cr	0.45 ± 0.34	<1	0.4 ± 1.3	1.4 ± 0.8	1.3 ± 1.76	2	0.89	**3.11**	**2.89**
THCs	1.31 ± 1.35	10	**3.5** ± **0.5**^**A**^	1.6 ± 0.2^B^	0.6 ± 0.2^C^	2	**2.67**	**1.22**	0.45

Values in tissue with different superscripts (A > B > C) are significantly different (p ≤ 0.05), while values with same superscript are not significantly different (p > 0.05). Emboldened concentrations in the tissues and water are higher than the established limits, while emboldened bioconcentration factors (BCF) are significant (≥1). (Confidence level = 95%). Sample size (n) = 288.

The concentration profile of trace metals and THCs in *Clarias gariepinus* (Fig. 2A) was: Fe (6.38%) > Zn (18.97%) > Mn (7.89%) > Cu (4.15%) > Cr (2.55%) > THC (0.81%) > Pb (0.2%) > Cd (0.05%). Meanwhile, the distribution of the toxicants in the tissues was in the order of gills (55%) > muscle (27%) > intestine (18%). The gills of *C. gariepinus* was most receptive to the toxicants, followed by the muscles, then the intestine (Fig. 2B).

Furthermore, in the gills, only the principal components 1 and 2 were above Eigenvalue 1 (Fig. 3I,A), hence were significant and considered for the plots in rotated space (Fig. 3II,A). The projection of toxicants in component 1 was in the order of Zn > Cd > THCs, while Fe > Cr > Pb > Mn projected towards component 2. However, Cu weakly spiked towards component 2, with some tendencies of affinity for component 1 (Fig. 3II,A).

In the intestines, the principal components 1, 2, and 3 were incorporated into the spatial rotation as they were the components of significant Eigenvalues in the intestine (Fig. 3I,B). In rotated space, Cd exhibited distinct affinity for component 2, while THCs > Fe > Zn also exhibited affinities for component 2 but they rather projected in the opposite direction to Cd (Fig. 3II,B). Conversely, Cu and Mn showed weak affinities for component 3, with some tendencies for the derived component 2. Although Cr and Pb were in proximity to the borderline between components 1 and 3, they however slightly spiked towards components 1 and 3 respectively (Fig. 3II,B).

Projection of Mn towards component 1 was weak, with some tendencies for component 2. The spike direction of Cd was consistent in both the intestine and muscles (towards component 2). Conversely, Pb > THCs > Cr had affinities for the derived component 2, while Fe > Cu exhibited marked affinities for component 3. Importantly, Zn occurred at the borderline between components 1 and 3 (Fig. 3II,C).

### Analysis of trace metals and THCs in *Clarias gariepinus* gills

#### Component analysis

Eigenvalues were determined empirically using detailed principal component analysis of concentrations of the trace metals and THCs in the gills of the fish. Results show that only percentage variance of initial Eigenvalues and extraction sum of squares of components 1(53.470%), followed by 2 (18.812%) were significant, amounting to cumulative percentages of 53.470% and 72.282% respectively (Table 2). The percentage variance of rotation sums of square were 47.550% and 24.733% for components 1 and 2 respectively, amounting to cumulative percentages of 47.550% and 72.282% respectively.

**Table 2:** Principal component analysis of C. *gariepinus* gill*s* using extraction method.

Component	Initial Eigenvalues			Extraction Sums of Square Loadings			Rotation Sums of Square Loadings		
	Total	% of Variance	Cumulative %	Total	% of Variance	Cumulative %	Total	% of Variance	Cumulative %
1	**4.278**	**53.470**	**53.470**	**4.278**	**53.470**	**53.470**	**3.804**	**47.550**	**47.550**
2	**1.505**	**18.812**	**72.282**	**1.505**	**18.812**	**72.282**	**1.979**	**24.733**	**72.282**
3	0.922	11.524	83.806						
4	0.571	7.139	90.945						
5	0.366	4.574	95.520						
6	0.292	3.653	99.173						
7	0.045	0.557	99.730						
8	0.022	0.270	100.000						

Legend: Emboldened values are significant^64–66,76^.

After the Principal component analysis (PCA) extraction, we employed direct oblimin with Kaiser Normalization in analyzing the 2 significant components earlier detected. The benchmark significant rotation components matrix (RCM) was set at 0.600. Results showed that the parameters with significant component matrices in component 1 were Pb (0.924) > Cr (0.920) > Fe (0.894) > Mn (0.793) > Zn (0.676) > Cd (0.666), while THCs was the only parameter with a significant RCM in component 2 (Table 3).

**Table 3:** Rotated component matrix.

Parameters	Components	
	1	2
Pb	**0.924**	−0.213
Cr	**0.920**	−0.001
Fe	**0.894**	0.040
Mn	**0.793**	−0.322
Zn	**0.676**	0.425
Cd	**0.666**	0.591
THCs	0.044	**0.689**
Cu	0.498	−0.592

Legend: Emboldened values are significant (≥0.6000).

#### Hierarchical cluster analysis (HCA) and correlation analysis

**Figure 4:**
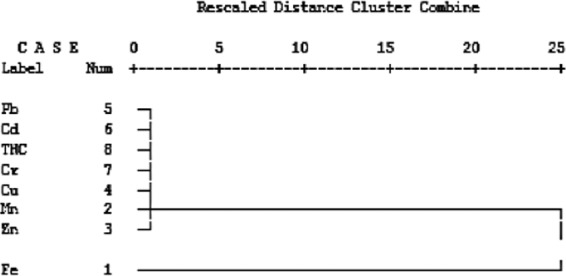
Dendrogram using average linkage (between groups), showing interrelatedness of toxicants’ activities in the gills.

Results of HCA showed Pb, Cd, THCs, Cr, Cu, Mn, and Zn were all clustered together, while Fe was distant from others by 25 units (Fig. 4). This implies that the concentration of iron in the gill of *C. gariepinus* did not correlate with other toxicants.

A significant negative correlation (−0.7432) occurred between Fe and Zn (Table 4). No significant correlation occurred between iron and other parameters analyzed. High positive correlations occurred between manganese and Cu (0.8045), Pb (0.7726), and Cr (0.8680). A high positive correlation also occurred between Pb and Cr (0.9298). Significant positive correlation also occurred between Zn and Pb (0.6630), Cu and Cr (0.6569). These results correspond with the results of the cluster analysis.

**Table 4:** Correlation of trace metals and THCs in gill of *Clarias gariepinus*.

	Fe	Mn	Zn	Cu	Pb	Cd	Cr	THCs
Fe	1							
Mn	0.1783	1						
Zn	**−0.7433**	0.4782	1					
Cu	**0.5581**	**0.8045**	0.3931	1				
Pb	**0.5670**	**0.7726**	**0.6630**	0.5640	1			
Cd	**0.5061**	0.2169	0.1940	−0.0433	0.1007	1		
Cr	0.1298	**0.8680**	0.43742	**0.6569**	**0.9298**	0.1980	1	
THCs	0.1156	0.1170	0.4737	0.0127	−0.1112	0.1607	0.0042	1

Emboldened values are significant (≥0.5) at *p* ≤ 0.05.

### Analysis of trace metals and THCs in *Clarias gariepinus* intestines

#### Component analysis

In the percentage variance of initial Eigenvalues and extraction sum of square, components 1(42.158%) > 2 (21.657%) > 3 (15.590%) were the major constituents, amounting to cumulative percentages of 42.158%, 63.815%, and 79.405% (Table 5). Percentage of variances of rotation sum of square were 35.090% > 27.898% > 16.416% which amounted to cumulative percentages of 35.090% <62.988% <79.405% for components 1, 2, and 3 respectively.

**Table 5:** Principal component analysis of *C. gariepinus* intestines using extraction method.

Component	Initial Eigenvalues			Extraction Sums of Squared Loadings			Rotation Sums of Squared Loadings		
	Total	% of Variance	Cumulative %	Total	% of Variance	Cumulative %	Total	% of Variance	Cumulative %
1	**3.373**	**42.158**	**42.158**	**3.373**	**42.158**	**42.158**	**2.807**	**35.090**	**35.090**
2	**1.733**	**21.657**	**63.815**	**1.733**	**21.657**	**63.815**	**2.232**	**27.898**	**62.988**
3	**1.247**	**15.590**	**79.405**	**1.247**	**15.590**	**79.405**	**1.313**	**16.416**	**79.405**
4	0.710	8.870	88.275						
5	0.484	6.046	94.321						
6	0.273	3.415	97.736						
7	0.110	1.380	99.116						
8	0.071	0.884	100.000						

Legend: Emboldened values are significant.

The PCA extractions were converged in three iterations using direct oblimin (with Kaiser Normalization) in analyzing the 3 significant components. Results showed that the parameters of significance in component 1 were Mn (0.894) > Cu (0.878) > Zn (0.792) > Fe (0.774) > Pb (0.669) were the significant toxicants in component 1. The order was Cr (−0.744) > Pb (−0.672) in component 2, while Cd (0.869) was the significant contaminant in component 3 (Table 6).

**Table 6:** Rotated component matrix.

Parameters	Component		
	1	2	3
Mn	**0.894**	0.012	0.073
Cu	**0.878**	0.061	0.009
Zn	**0.792**	0.442	0.314
Fe	**0.600**	0.519	0.060
Cr	0.547	**−0.744**	−0.134
Pb	**0.669**	**−0.672**	−0.134
Cd	−0.052	−0.074	**0.869**
THCs	0.254	0.503	−0.591

Legend: Emboldened values are significant (≥0.6000).

#### Hierarchical cluster and correlation analyses

**Figure 5:**
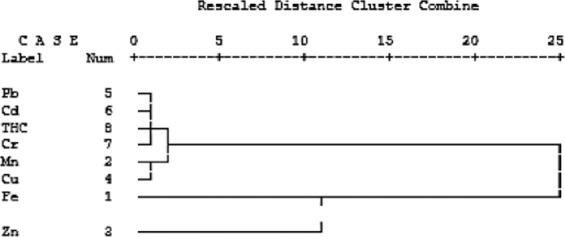
Dendrogram using average linkage (between groups), showing interrelatedness of toxicants’ activities in the intestines.

Results of HCA showed Pb, Cd, THCs, and Cr formed a cluster. In close association with this cluster is another cluster formed between Mn and Cu. In this cluster, Pb, Cd, THCs, and Cr formed a sub-cluster and Mn and Cu formed another sub-cluster. Iron and Zn occurred as independent pollutants and in isolation in the intestine dendogram (Fig. 5).

The correlation results (Table 7) of trace metals and THCs in the intestine of *C. gariepinus* conform to the results of the dendogram. A significant correlation occurred between Fe and Zn (0.63327), and a highly significant correlation occurred between Mn and Cu (0.89189). There were moderate but significant correlations between Cu and Cr (0.65685). Lead also exhibited high correlations with Cr (0.92979), and THCs (0.71123). Cadmium concentration also highly correlated with chromium (0.72126). THCs had significant correlations with contaminants in same dendogram cluster i.e. Pb (0.71123), Cd (0.6542), and Cr (0.6622).

**Table 7:** Correlations of trace metals and THCs in intestines of *Clarias gariepinus*.

	Fe	Mn	Zn	Cu	Pb	Cd	Cr	THCs
Fe	1							
Mn	0.3333	1						
Zn	**0.6333**	0.3578	1					
Cu	0.4581	**0.8919**	0.1921	1				
Pb	0.3211	0.4311	0.1621	0.1133	1			
Cd	0.3022	**0.5019**	0.2310	−0.0433	0.1221	1		
Cr	0.2123	0.1672	0.4117	**0.6569**	**0.9298**	**0.7213**	1	
THCs	0.3112	0.1070	**0.5127**	0.0111	**0.7113**	**0.6542**	**0.6622**	1

Emboldened values are Significant (≥0.5) at *p* ≤ 0.05.

### Analysis of the trace metals and THCs in *Clarias gariepinus* muscles

#### Component analysis

The PCA results showed similar values for percentage variance of initial Eigen and extraction sum of squares for components 1(36.842) > 2 (22.531) > 3 (15.671) which were significant; as their Eigenvalues were above 1 (Fig. 2I,C). These amounted to cumulative percentages of 36.842, 59.374, and 75.045 respectively (Table 8). The percentage of variance of rotation sum of square for Components 1 (33.864) > 2 (24.351) > 3 (16.830) amounted to cumulative percentages of 33.864% < 58.215% < 75.045% respectively.

**Table 8:** Principal component analysis of *C. gariepinus* muscles using extraction method.

Component	Initial Eigenvalues			Extraction Sums of Squared Loadings			Rotation Sums of Squared Loadings		
	Total	% of Variance	Cumulative %	Total	% of Variance	Cumulative %	Total	% of Variance	Cumulative %
1	**2.947**	**36.842**	**36.842**	**2.947**	**36.842**	**36.842**	**2.709**	**33.864**	**33.864**
2	**1.803**	**22.531**	**59.374**	**1.803**	**22.531**	**59.374**	**1.948**	**24.351**	**58.215**
3	**1.254**	**15.671**	**75.045**	**1.254**	**15.671**	**75.045**	**1.346**	**16.830**	**75.045**
4	0.994	12.420	87.465						
5	0.424	5.306	92.771						
6	0.312	3.903	96.674						
7	0.235	2.938	99.612						
8	0.031	0.388	100.000						

Legend: Emboldened values are significant.

Furthermore, PCA extractions were converged in three iterations using direct oblimin (with Kaiser Normalization) in analyzing the 3 significant components. Results showed that the parameters of significance in Component 1 were in the order of Cr (0.888) > Cu (0.836) > THCs (0.668) > Mn (0.626) > Fe (0.621), the order in component 2 was Zn (−0.705) > Pb (0.700) > Fe (−0.665), while component 3 was Cd (0.776) only (Table 9).

**Table 9:** Rotated component matrix.

Parameters	Component		
	1	2	3
Cr	**0.888**	0.136	−0.227
Cu	**0.836**	0.124	0.251
THCs	**0.668**	0.238	−0.106
Mn	**0.626**	0.523	0.341
Zn	0.334	**−0.705**	−0.314
Pb	0.241	**0.700**	−0.525
Fe	**0.621**	**−0.665**	−0.191
Cd	0.256	**−0.092**	**0.776**

Emboldened values are Significant (≥0.6000).

#### Hierarchical cluster analysis

**Figure 6:**
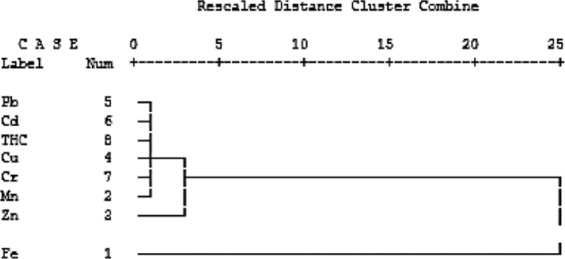
Dendrogram using average linkage (between groups), showing interrelatedness of toxicants’ activities in the muscles.

In the muscles, the HCA showed that Pb, Cd, THCs, Cu, Cr, and Mn formed a cluster, which was distant from Zn, and further distant from Fe, which was distinctively isolated from others (Fig. 6). The dendogram of contaminants in the muscle tissue of *C. gariepinus* was buttressed by results of the correlation (Table 10). Significant correlations occurred among contaminants found in the same dendogram cluster i.e. manganese significantly correlated with copper (0.64045), lead (0.67257), cadmium (0.63168), and THCs (0.64704). Copper had significant correlation with Cd (0.6334), and Cr (0.6569). Lead exhibited a significant relationship with cadmium (0.7238) and chromium (0.9298). A highly significant correlation (0.83802) also occurred between cadmium and chromium.

**Table 10:** Correlations of trace metals and THCs in the muscles of *Clarias gariepinus*.

	Fe	Mn	Zn	Cu	Pb	Cd	Cr	THCs
Fe	1							
Mn	0.1783	1						
Zn	0.3327	0.3122	1					
Cu	0.4481	**0.64045**	0.3931	1				
Pb	0.2167	**0.67257**	0.3630	0.4440	1			
Cd	0.3061	**0.63168**	0.5922	**0.6334**	**0.7238**	1		
Cr	0.1298	0.36804	**0.7474**	**0.6569**	**0.9298**	**0.8380**	1	
THCs	0.1156	**0.64704**	0.2724	**0.7127**	**0.8311**	0.7616	**0.6332**	1

Emboldened values are Significant (≥0.5) at *p* ≤ 0.05.

Of high importance is the fact that THCs had significant correlations with all other contaminants in the same dendogram cluster. No correlations occurred between Fe and Zn and any other toxicant analyzed in the muscle.

## Discussions

High level of variability which occurred in the contaminant profile across the tissues of *C. gariepinus* is a function of uptake route, varied organ functions and specificity of tissue permeability of the compared organs^63^. However, iron being dissociated from other contaminants in the dendogram of the gills and being associated with same contaminants in dendogram of the intestine showed that organ-specificity dominated contaminant bioavailability among factors that influenced accumulation in the fish^64–66,67,68^.

The gill of a fish is an organ which is always in contact with the water medium, hence has the highest level of exposure to the contaminants in the aqueous phase. The concentrations of Fe and THCs in the fish gill were higher than FEPA limits. Iron’s dissociation from other metals accompanied low concentrations of Zn and Mn as expected. High concentrations of iron can be linked to its high threshold of essentiality in the hemoglobin, required for respiration in the gills.

The dendogram analysis further showed that the absorption of Fe in the gills varied appreciably compared to that of other contaminants (Pb, Cd, THCs, Cr, Cu, Mn, and Zn) among which high conformity occurred. This was supported by the significant negative correlation which occurred between Fe and most of the contaminants analyzed which indicates that iron inhibited accumulation of the other trace metals in the gills owing to its indispensable threshold of essentiality in the organ. Inverse relationship of Fe concentration with Zn absorption has previously been reported^14,62^. Previous studies have also shown an inverse relationship between hepatic Mn and Fe concentrations^6,19,69^. This is due to the fact that manganese and iron transport is regulated by transferrin receptor and divalent metal transporter. Hence, competition for binding sites may occur.

In the gills of the fish, the actual order of contamination was Pb > Cr > Fe > Mn in principal component 1. Occurrence of iron as an active contaminant in principal component 1 of the gills is attributable to its function as an active component of hemoglobin during respiration. Although concentration of Pb in the gill was lower than the FEPA limit, it was significantly higher than the concentrations in other tissues. Furthermore, the concentration was not far below the regulatory limit. This implies that persistent bioaccumulation may be a prognosis of hazardous concentration of Pb. Lead being the leading contaminant in the gill may elicit teratogenic effects in the fish. Lead poisoning may also cause inhibition of the synthesis of hemoglobin, dysfunctions in the kidneys, bone joints, reproductive systems, cardiovascular system, and damage to the nervous system^5,20^. The concentration of THCs in the gills exceeded the FEPA limit, implying that teratogenic, mutagenic and carcinogenic effects may ensue^24,70^. The association of THCs with most of the trace metals in the dendogram indicates that the toxicants might have been released from same source which is attributable to the petroleum activities of the oil company, considering evidences previously provided by Imoobe and Adeyinka^55^, Isibor^31^, and Isibor & Imoobe^47^.

Comparison of accumulation among the tissues showed that the gills accumulated the most toxicants, particularly Fe which had a markedly high BCF of 145.44 (>1) and actual concentration (181.8 ± 42.3 mg/kg) greater than the established limit (100 mg/kg). Iron was also one of the most active toxicants in the tissue. If unregulated in the aquatic habitat, excess iron may elicit tissue injury, characterized by gill tissue necrosis, impairment of the excretory function of the gills and elevation of transaminases activities in the blood plasma of the fish. The gills being an unpalatable part of the fish is discarded before processing in some Nigerian homes. A few restaurants also process the whole fish and discard the unwanted parts, including the gills and internal organs, prior to cooking. Thus, these contaminants may not be transferred into the entire food in such cases. Conversely however, many restaurants process the whole fish without discarding the unwanted and toxicologically implicated organs. Such process may expose the consumers to Fe toxicity which may result in multi-system organ failures, coma, convulsion and ultimately death in extreme cases ^51,71^. The outstandingly high concentration of the contaminants detected in the gills is attributable to the organ’s physiological and anatomical properties that maximize absorption efficiency. The organ also outstandingly plays physiological roles of iono-regulation, osmoregulation and respiration; hence interacts more with the toxicants in the water than the intestines and muscles.

In the intestine, results of communality extractions and rotated component matrix revealed that the order of contaminants was Mn > Cu > Zn in component 1. The trend is the reverse of the observations of Omar *et al*.^72^ and Silva *et al*.^62^ in *Paralichthys brasiliensis*. Zinc plays an essential role as an element required for metabolic digestion in the intestine. Fish is a good source of Zn which is a trace element in biological systems. Zinc is one of the essential elements for the metabolism of carbohydrates, proteins, lipids, and nucleic acids. However the concentration of Zn detected in the intestine far exceeded the concentrations in other organs investigated. It also exceeded the set standard of FEPA^60^ by a margin of 13.9 mg/kg wt w. Excess Zn in diet may cause immunosuppressive effects, thereby reducing the stimulation responses of lymphocytes and alterations in cholesterol metabolism^27^. Its deficiency in the body can lead to loss of appetite, growth retardation, and immunological problems^40^. Antagonistic relationship between Zn and Fe observed in this study corroborates the observations in previous studies^6,7,19,26,50^.

The results of communality extractions and rotated component matrix showed the order of contaminants in the muscles was Cr > Cu in component 1; and Zn and Pb in component 2. Investigations also showed that Mn (6.5 mg/kg wtw) and Cu (4.6 mg/kg wt w) in the muscles were significantly higher than the concentrations observed in the other organs and the FEPA^60^ limits which are 1 mg/kg and 3 mg/kg respectively. The high concentrations of Cu in the muscles are attributable to its high bioconcentration factor. Its high concentrations in the muscle of the fish which is the major edible part^58^ may expose the consumers of the fish to risks of liver cirrhosis^72^ because essential elements may be toxic when in excess^47^. Similarly, high concentration of Cu (33.7 μg g^−1^) was detected in carapeba (*Diaptereus rhombeus*) by Silva *et al*.^62^. Similar concentrations have also been detected in muscles of other freshwater teleosts^33,63,73^ and marine species^62^.

Higher accumulation of the toxicants in the muscles than the intestine is at variance with the expected order of gills > intestines > muscles^74^. Higher accumulation was expected in the intestines than the muscle because the intestine is more metabolically active. Moreover, *Clarias gariepinus* being a bottom feeder is expected to readily accumulate contaminants in its intestine from the repository riverbed. Similar deviant order was earlier observed by Aderinola *et al*.^26^ in *Chrysichthys nigrodigitatus, Tilapia zillii* and *Macrobrachium macrobrachion* of Badagry creek in Lagos State, Nigeria, while Ekeanyanwu *et al*.^75^ observed same in *Chrysichthys nigrodigitatus* and *Tilapia nilotica* of Okumeshi river in Delta State, Nigeria.

Manganese toxicity effects occur mainly in the respiratory and nervous systems. Symptoms of manganese poisoning are mainly hallucinations, forgetfulness characterized by poor cognitive performance and nerve damage. Manganese can also cause Parkinson’s disease, lung embolism, bronchitis, schizophrenia, dullness, weak muscles, headaches and insomnia^20^. Prolonged exposure of the male sex organ to Mn may result in sterility and impotence^73^.

Manganese and copper being the most bioconcentrated toxicants in the muscle of the investigated fish and at the same time Cu being of excess concentrations in the tissue warrant a stringent remediation of the aquatic habitat. This is due to the fact that the muscle is the major edible part of fish and constitutes the major part of its body weight^58^; hence holds its nutritional and economic values^21^.

## Conclusions

High variability in the toxicant profile across the tissues of *C. gariepinus* is a function of uptake route, varied organ functions and specificity of tissue permeability of the compared organs. The study demonstrated variability in organ accumulation capacity and toxicant’s competitiveness irrespective of bioavailability. The study provides data useful for future ecotoxicological studies and safety of consumers of the *C. gariepinus* in Osse River. A constant check on the river through integrated biomonitoring studies using sentinel species such as the plankton and benthic organisms are promising tools which may contribute effectively to the information on the condition of the aquatic habitat. In the meantime, removal of the gills and intestines of the fish before processing for consumption is recommended.

## Supplementary information

Supplementary Data.
