# Supplementary material for: Principal Components and Hierarchical Cluster Analyses of Trace Metals and Total Hydrocarbons in Gills, Intestines and Muscles of Clarias gariepinus (Burchell, 1822)
Source: Sci Rep. 2020 Mar 20;10:5180. doi: 10.1038/s41598-020-62024-9 (PMC7083867; doi:10.1038/s41598-020-62024-9)
Supplement: Supplementary file 1 — Supplementary Data. [file 41598_2020_62024_MOESM1_ESM.docx]

**Principal Components and Hierarchical Cluster Analyses of Trace Metals and Total Hydrocarbons in Gills, Intestines and Muscles of *Clarias gariepinus* (Burchell, 1822)**

**^1^ Patrick Omoregie Isibor*, ^2^ Tunde, O. Thaddeus Imoobe, ^2^Alex Ajeh Enuneku, ^1^Paul Akinniyi Akinduti, ^3^Gabriel Adewunmi Dedeke, ^4^Theophilus Aanuoluwa Adagunodo, and ^1^Dorcas, Yemisi Obafemi**

^1^Department of Biological Science, College of Science and Technology, Covenant University, Ota, Ogun State, Nigeria*

^2^Department of Animal and Environmental Biology, University of Benin, Nigeria.

^3^Department of Pure and Applied Zoology, College of Bioscience, Federal University of Agriculture, Abeokuta, Ogun State, Nigeria.

^4^Department of Physics, College of Science and Technology, Covenant University, Ota, Ogun State, Nigeria

**RAW DATA OF WATER PHYSICOCHEMICAL PARAMETRS**

| Years | Months | Mg (mg/l) | Cl (mg/l) | P (mg/l) | NO3 (mg/l) | SO4 (mg/l) | Fe (mg/l) | Mn (mg/l) | Zn (mg/l) | Cu (mg/l) | Cr (mg/l) | Cd (mg/l) | Pb  (mg/l) | V  (mg/l) | THC  (mg/l) |
| --- | --- | --- | --- | --- | --- | --- | --- | --- | --- | --- | --- | --- | --- | --- | --- |
| 2013 | Apr | 1.2 | 124.1 | 1.39 | 0.21 | 1.27 | 2.45 | 2.12 | 3.27 | 2.12 | 1.24 | 1.15 | 2.66 | 0.02 | 2.33 |
|  | May | 0.69 | 177.3 | 2.48 | 0.01 | 7.49 | 5.41 | 2.34 | 0.46 | 1.23 | 1.12 | 1.33 | 2.46 | 0.033 | 0.11 |
|  | Jun | 1.55 | 53.2 | 0.89 | 0.05 | 2.1 | 1.35 | 2.68 | 1.43 | 2.33 | 0.06 | 0.05 | 2.22 | 0.027 | 0.08 |
|  | Jul | 0.12 | 28.8 | 2.88 | 2.11 | 3.94 | 1.06 | 0.11 | 0.6 | 2.12 | 0.05 | 0.01 | 0.01 | 0.08 | 1.85 |
|  | Aug | 0.07 | 16.5 | 1.65 | 1.21 | 2.26 | 0.61 | 0.06 | 0.35 | 2.11 | 2.22 | 0 | 0.01 | 0.02 | 2.34 |
|  | Sep | 0.05 | 12.3 | 1.23 | 0.9 | 1.68 | 1.27 | 0.05 | 0.08 | 0.04 | 0.2 | 0 | 0.01 | 0.01 | 2.45 |
|  | Oct | 0.02 | 6 | 0.6 | 0.44 | 0.82 | 0.62 | 0.02 | 0.04 | 0.02 | 0.01 | 0 | 0 | 0.01 | 0.85 |
|  | Nov | 0.04 | 9.3 | 0.93 | 0.68 | 1.27 | 0.22 | 0.03 | 0.06 | 0 | 0.02 | 0 | 0 | 0 | 0 |
|  | Dec | 0.05 | 13.2 | 1.32 | 0.97 | 1.8 | 0.25 | 0.05 | 0.09 | 0 | 0 | 0 | 0.01 | 0 | 0 |
| 2014 | Jan | 0.05 | 11.4 | 1.14 | 0.84 | 1.56 | 1.18 | 0.04 | 0.08 | 0 | 0 | 0 | 0.01 | 0 | 0 |
|  | Feb | 0.04 | 12.6 | 1.34 | 1.3 | 1.7 | 1.22 | 0.03 | 0.21 | 0.02 | 0 | 0.02 | 0.01 | 0 | 0.05 |
|  | Mar | 0.05 | 13.2 | 1.32 | 0.97 | 1.8 | 1.36 | 0.05 | 0.09 | 0.04 | 0.13 | 0 | 0.01 | 0 | 0.09 |
|  | Apr | 0.07 | 18.6 | 1.86 | 1.36 | 2.54 | 1.92 | 0.07 | 0.12 | 0.06 | 0.19 | 0.02 | 0.04 | 0.03 | 0.11 |
|  | May | 0.78 | 3.33 | 3.33 | 2.44 | 3.79 | 1.44 | 3.22 | 2.22 | 2.33 | 2.11 | 0.05 | 2.22 | 0.09 | 2.33 |
|  | Jun | 0.23 | 12.5 | 3.2 | 2.5 | 3.22 | 1.23 | 3.56 | 2.32 | 2.21 | 2.33 | 0.03 | 0.83 | 0.08 | 2.22 |
|  | Jul | 0.12 | 13.5 | 3.3 | 2.6 | 4.52 | 1.12 | 3.67 | 1.86 | 2.76 | 1.23 | 1.15 | 2.11 | 0.07 | 2.13 |
|  | Aug | 0.23 | 14.8 | 1.08 | 2.8 | 8.83 | 1.04 | 2.22 | 2.13 | 1.21 | 1.11 | 1.11 | 2.12 | 0.06 | 3.23 |
|  | Sep | 0.44 | 13.8 | 1.3 | 3.1 | 7.44 | 1.12 | 2.33 | 2.32 | 0.22 | 1.12 | 2.34 | 0.12 | 0.04 | 2.45 |

Correlation of water parameters


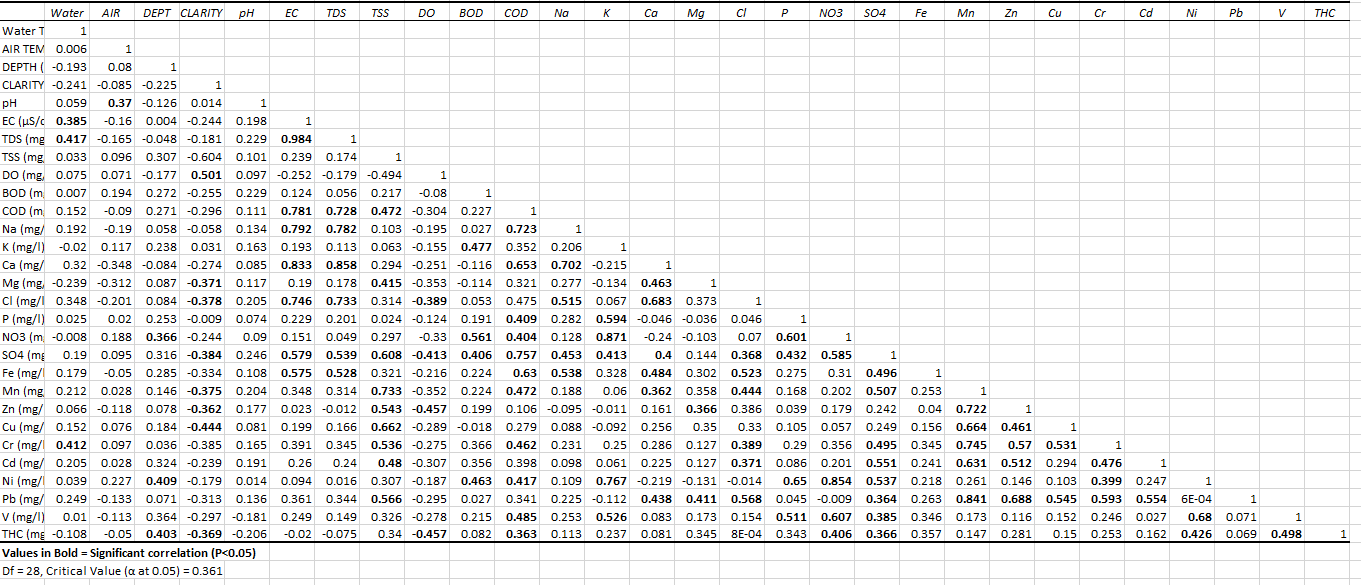


**RAW DATA OF PARAMETERS (mg/kg) in *Clarias gariepinus***

| Tissues | Years | Months | Fe | Mn | Zn | Cu | Pb | Cd | Cr | THC |
| --- | --- | --- | --- | --- | --- | --- | --- | --- | --- | --- |
| Gills | 2013 | Apr | 120.7 | 1.68 | 78.62 | 2.24 | 0 | 0.09 | 0.06 | 5.2 |
|  |  | May | 131.8 | 2.1 | 70.1 | 3.09 | 0 | 0.11 | 0.06 | 6.2 |
|  |  | Jun | 202.75 | 13.55 | 60.54 | 10.2 | 0 | 0.08 | 0.01 | 7.25 |
|  |  | Jul | 375.58 | 10.76 | 54.34 | 18.43 | 1.78 | 0.11 | 11.08 | 8.2 |
|  |  | Aug | 233.76 | 26.49 | 43.12 | 11.01 | 1.14 | 0.1 | 6.75 | 5.8 |
|  |  | Sep | 638.76 | 58.2 | 117.82 | 15.9 | 3.12 | 0.28 | 18.45 | 3.45 |
|  |  | Oct | 503.76 | 45.9 | 92.92 | 12.54 | 2.46 | 0.22 | 14.55 | 2.11 |
|  |  | Nov | 658.65 | 45.8 | 54.55 | 10.5 | 1.53 | 0.01 | 12.55 | 3.8 |
|  |  | Dec | 22.45 | 12.5 | 12.55 | 6.88 | 0.5 | 0 | 0.01 | 1.32 |
|  | 2014 | Jan | 21.53 | 5.08 | 10.32 | 2.35 | 0.01 | 0.01 | 0.55 | 0.56 |
|  |  | Feb | 244.13 | 6.99 | 35.32 | 11.98 | 1.16 | 0.07 | 7.2 | 1.23 |
|  |  | Mar | 302.72 | 8.67 | 43.8 | 14.85 | 1.43 | 0.09 | 8.93 | 1.41 |
|  |  | Apr | 190.71 | 5.46 | 27.59 | 9.36 | 0.9 | 0.06 | 5.63 | 2.83 |
|  |  | May | 202.55 | 13.55 | 85.22 | 22.56 | 0.8 | 0.01 | 5.3 | 2.82 |
|  |  | Jun | 350.42 | 12.45 | 78.45 | 15.34 | 0.6 | 0.02 | 4.65 | 1.22 |
|  |  | Jul | 280.5 | 62.32 | 85.55 | 33.68 | 1.34 | 0.11 | 6.75 | 3.82 |
|  |  | Aug | 355.45 | 45.87 | 56.55 | 45.56 | 2.1 | 0.02 | 6.89 | 2.05 |
|  |  | Sep | 235.56 | 55.5 | 33.32 | 22.35 | 1.8 | 0.01 | 5.43 | 3.6 |
| Intestine | 2013 | Apr | 98.23 | 1.11 | 40.18 | 0.6 | 0 | 0.06 | 0.04 | 2.1 |
|  |  | May | 91.9 | 1.83 | 56.8 | 1.9 | 0 | 0.07 | 0.05 | 2.45 |
|  |  | Jun | 92.77 | 3.55 | 55.6 | 12.56 | 0.01 | 0.05 | 0 | 3.12 |
|  |  | Jul | 59.91 | 9.53 | 13.86 | 6.45 | 0.43 | 0.06 | 11.36 | 2.56 |
|  |  | Aug | 85.84 | 36.28 | 66.42 | 10.83 | 0.21 | 0.06 | 4.91 | 3.45 |
|  |  | Sep | 153.04 | 37.35 | 118.82 | 19.31 | 0.38 | 0.11 | 8.75 | 0.65 |
|  |  | Oct | 53.59 | 13.08 | 41.46 | 6.76 | 0.13 | 0.04 | 3.07 | 0.75 |
|  |  | Nov | 45.77 | 4.08 | 40.5 | 4.32 | 0.14 | 0.56 | 3.23 | 0.33 |
|  |  | Dec | 35.01 | 2.34 | 11.55 | 2.22 | 0 | 0 | 0.01 | 0.65 |
|  | 2014 | Jan | 15.53 | 1.55 | 8.55 | 1.45 | 0.01 | 0 | 0.58 | 0.76 |
|  |  | Feb | 38.94 | 6.19 | 9.01 | 4.19 | 0.28 | 0.04 | 7.38 | 0.96 |
|  |  | Mar | 48.29 | 7.68 | 11.17 | 5.2 | 0.35 | 0.05 | 9.16 | 1.19 |
|  |  | Apr | 30.42 | 4.84 | 7.04 | 3.28 | 0.22 | 0.03 | 5.77 | 0.75 |
|  |  | May | 70.55 | 5.58 | 18.45 | 5.56 | 0.21 | 0.04 | 3.5 | 2.22 |
|  |  | Jun | 88.5 | 6.87 | 20.55 | 12.5 | 0.33 | 0.02 | 4.5 | 1.55 |
|  |  | Jul | 67.85 | 8.65 | 35.78 | 10.8 | 0.24 | 0.01 | 5.2 | 2.42 |
|  |  | Aug | 77.56 | 10.2 | 45.55 | 15.45 | 0.11 | 0.03 | 6.5 | 1.35 |
|  |  | Sep | 35.32 | 28.75 | 98.56 | 20.5 | 0.41 | 0.02 | 4.54 | 1.8 |
| Muscle | 2013 | Apr | 116.1 | 1.34 | 65.93 | 1.89 | 0 | 0.09 | 0.06 | 0.23 |
|  |  | May | 220.1 | 2.78 | 83.2 | 3.65 | 0 | 0.13 | 0.13 | 0.33 |
|  |  | Jun | 180.03 | 15.07 | 75.5 | 1.05 | 0 | 0.45 | 0 | 0.42 |
|  |  | Jul | 31.07 | 35.11 | 31.87 | 1.98 | 0.34 | 0.15 | 4.68 | 1.2 |
|  |  | Aug | 142.39 | 29.19 | 37.73 | 7.97 | 0.23 | 0.09 | 13.53 | 2.1 |
|  |  | Sep | 242.39 | 25.45 | 64.23 | 13.57 | 0.39 | 0.15 | 23.03 | 2.3 |
|  |  | Oct | 182.39 | 19.15 | 48.33 | 10.21 | 0.29 | 0.11 | 17.33 | 0.2 |
|  |  | Nov | 88.52 | 33.78 | 35.7 | 13.57 | 0.001 | 1.56 | 8.67 | 1.2 |
|  |  | Dec | 23.05 | 12.22 | 8.56 | 1.05 | 0 | 0 | 0.02 | 0.7 |
|  | 2014 | Jan | 20.2 | 5.65 | 7.23 | 2.13 | 0.23 | 0 | 0.66 | 0.3 |
|  |  | Feb | 20.2 | 22.82 | 20.72 | 1.29 | 0.22 | 0.1 | 3.04 | 0.21 |
|  |  | Mar | 25.04 | 28.3 | 25.69 | 1.6 | 0.27 | 0.12 | 3.77 | 0.26 |
|  |  | Apr | 15.78 | 17.83 | 16.18 | 1.01 | 0.17 | 0.08 | 2.38 | 0.16 |
|  |  | May | 156.45 | 17.55 | 18.55 | 12.4 | 0 | 0.06 | 4.2 | 0.28 |
|  |  | Jun | 187.54 | 30.25 | 20.56 | 12.66 | 0.01 | 0.03 | 6.6 | 0.32 |
|  |  | Jul | 220.4 | 32.45 | 25.55 | 11.45 | 0.01 | 0.03 | 8.85 | 0.3 |
|  |  | Aug | 105.68 | 29.5 | 35.55 | 10.67 | 0.22 | 0.01 | 14.5 | 0.2 |
|  |  | Sep | 125.5 | 28.24 | 65.68 | 10.5 | 0 | 0.02 | 20.5 | 0.66 |
